# The effect of aromatherapy on post-stroke depression: study protocol for a pilot randomized controlled trial

**DOI:** 10.3389/fpsyt.2024.1428028

**Published:** 2024-07-25

**Authors:** Yujia Li, Zekai Hu, Kun Zhou, Yanyu Wang, Xinglin Zhang, Han Xue, Jun Hu, Jie Wang

**Affiliations:** ^1^ School of Rehabilitation Science, Shanghai University of Traditional Chinese Medicine, Shanghai, China; ^2^ Traditional Chinese Medicine Rehabilitation Department, The Second Rehabilitation Hospital of Shanghai, Shanghai, China; ^3^ Department of Rehabilitation Medicine, Shanghai Zhongye Hospital, Shanghai, China

**Keywords:** post-stroke depression, cognitive behavior therapy, aromatherapy, depression, anxiety

## Abstract

**Background:**

Post-stroke depression (PSD) is a prevalent psychiatric disorder affecting about one-third of stroke survivors, significantly hindering recovery and quality of life. PSD also imposes a substantial burden on caregivers and healthcare systems. Aromatherapy has shown promise in alleviating depression, anxiety, and sleep disorders. This pilot randomized controlled trial aims to assess the feasibility and preliminary efficacy of mixed herb aromatherapy in treating PSD. Feasibility outcomes encompass recruitment, intervention adherence, assessment completion and safety assessment. Secondary outcomes include evaluations of depression, anxiety, cognitive function, sleep quality, quality of life, and brain function using EEG and fNIRS.

**Methods:**

This single-blind pilot randomized controlled trial will be conducted at the Second Rehabilitation Hospital of Shanghai, enrolling ninety-nine post-stroke patients with PSD. Participants will be randomized into three groups: a Non-Active Control Group receiving standardized rehabilitation therapy, a CBT Group receiving conventional rehabilitation with bi-weekly CBT sessions, and an Aromatherapy Group receiving conventional rehabilitation with daily aromatic inhalation sessions. Interventions will last for four weeks, with efficacy assessed at baseline, post-intervention, and one month post-intervention. Rating scales will be used to measure changes in depression, sleep quality, cognitive function, and quality of life. EEG and fNIRS will specifically be used to measure changes in cerebral cortex activity and their correlations with depression. Feasibility will be evaluated through recruitment, intervention adherence, assessment completion and safety assessment.

**Discussion:**

This pilot study highlights the potential of mixed herb aromatherapy inhalation for treating PSD, addressing limitations of CBT by promoting self-management. While demonstrating feasibility through recruitment, adherence, assessment completion and safety assessment, the study also acknowledges limitations such as unequal intervention times, the lack of physical function data. And the use of culturally relevant plant powders may enhance compliance but limits generalizability. Despite these constraints, the study provides valuable preliminary data and insights into the mechanisms of aromatherapy, encouraging further research and development of effective PSD treatments.

## 1 Introduction

### 1.1 Background and rationale

Stroke is a cerebrovascular disease characterized by high morbidity, disability, mortality, recurrence and economic burden, and has become the second leading cause of death and major disability worldwide (1). Post-stroke depression (PSD) is one of the most frequent psychiatric disorders in post-stroke patients, which seriously affects the overall recovery process, functional outcome, and quality of life of the patients, and increases the burden on their caregivers, as well as poses a great challenge to medical and social resources. The overall prevalence of PSD ranges from 11% to 41% due to differences in diagnostic criteria, sample sizes, geographic locations, and patient conditions (2), and deserves widespread attention.

The clinical manifestations of PSD involve multiple symptoms in the emotional, cognitive, physiological, and psycho-motor domains, including: anxiety, irritability, nervousness, sadness, disinterest, feelings of hopelessness, dulled sensation, attention deficit, low self-appraisal, sleep disorders, fatigue and weakness, loss of appetite, pain, restlessness, and motor retardation (3).

Researchers have summarized the risk factors for PSD, which include: genetics, gender, age, history of depression prior to stroke, impaired cognitive and motor function, stroke severity, lesion site, marital status, years of education, and social support (2, 4, 5). Some researchers have suggested that stroke increases the risk of PSD and depression is an independent risk factor for stroke and stroke-induced death, so the bidirectional relationship complicates the diagnosis and management of PSD (6). Currently, the pathophysiological mechanisms of PSD are unclear and may be related to neurobiological, behavioral, and social factors. Feng et al. (7) pointed out that neurobiological changes are most specific to PSD and are distinct from other subtypes of depression. Therefore, most studies have focused on neurobiological changes after stroke. There are various hypotheses in the academic community, such as the lesion site hypothesis, the neurotransmitter hypothesis, and the hypothalamic-pituitary-adrenal (HPA) axis dysfunction hypothesis (2, 7, 8).

The treatment of PSD is a comprehensive process. In addition to medication and psychotherapy, which are the two traditional treatments for PSD, modern rehabilitation therapy (physical therapy, occupational therapy, etc.) and Traditional Chinese medicine (TCM) rehabilitation therapies also play their respective advantages in PSD treatment. Cognitive behavioral therapy (CBT) is one of the most commonly used psycho-therapeutic methods for treating depression, which transforms patients’ negative ways of thinking and undesirable behaviors into positive coping skills. Wang et al. (9) found through a meta-analysis that CBT was effective in improving anxiety, neurological deficits, and daily life activities in PSD patients. A study on CBT for post-stroke fatigue and sleep disturbance found that CBT significantly reduced fatigue compared to treatment as usual (10). The study also discovered that CBT improved sleep quality and depression, with effects lasting two months post-therapy (10). These findings support CBT as an effective treatment for PSD. However, despite its efficacy, CBT has certain limitations. It requires significant time and commitment from patients, which can be challenging for those with severe depression or cognitive impairments. Access to trained CBT therapists may be limited in some areas, making it difficult for some patients to receive consistent treatment. Additionally, CBT sessions can be costly and may not be covered by all insurance plans. Given these challenges, our research aims to investigate alternative therapeutic options that may overcome these barriers.

Aromatherapy is a medical treatment method involving the use of fragrant plant extracts, typically essential oils, applied through inhalation, transdermal absorption, or oral administration to prevent and treat diseases (11). Aromatherapy offers several advantages over traditional therapies like CBT. It can be self-administered, encouraging patients to take an active role in their own care and self-management. This form of therapy does not necessarily require the presence of a professional therapist, making it more accessible and potentially more cost-effective. Furthermore, aromatherapy can easily be integrated into daily routines, providing continuous therapeutic benefits (12).

Aromatherapy has a long history. As early as about 2800 BC, the ancient Egyptians used resins, balsams, and aromatic oils to prevent and treat disease, and the ancient Egyptian scholar Papyrus Ebers wrote a famous manuscript on aromatic medicines (13). Also in 2800 B.C., the ancient text “Shennong’s manuscript” lists 350 plant species used in TCM. The “Yellow Emperor’s Classic of Internal Medicine” (HUNDI NEIJING) mentions that “aromatherapy,” also known as “moxibustion therapy,” can treat illnesses. This TCM theory holds that aromas and medicines share a common origin. The volatile compounds produced by aromatic herbs are believed to have therapeutic effects on the human body, aiding in the prevention and treatment of diseases.

Numerous studies have shown that aromatherapy is effective in improving depressive symptoms in a number of areas, including depression, anxiety, and sleep disturbance. For instance, a meta-analysis (14) demonstrated that aromatherapy using different essential oils significantly alleviated anxiety, regardless of the cause, though further research is needed to determine optimal dosages. Hui-Yue Lin et al. (15) studied the therapeutic effects of aromatics on depression and discovered that clove essential oil nebulization was effective in treating depression. Additionally, several studies (16, 17) have shown that aromatherapy can positively impact sleep quality. TCM scholars have also reviewed aromatic herbs for insomnia treatment, identifying herbs like Ligusticum chuanxiong and rose for blood circulation and qi movement; Cyperus rotundus, Albiziae flos, and bergamot for liver soothing and qi movement; Acori tatarinowii rhizome for dampness dissolution and mind calming when paired with other herbs; and lavender, Matricaria chamomilla L., and Valeriana officinalis L. for mind calming (18). However, while the efficacy of aromatherapy in relieving depression is well-supported, specific studies on its impact on PSD are limited. Recent studies have demonstrated the potential of aromatherapy in alleviating symptoms of depression and improving sleep quality in PSD patients. For instance, a study found that inhalation of lavender essential oil effectively reduced depression and improved sleep quality in PSD patients, indicating that aromatherapy could be a promising adjunctive treatment for PSD (19).

However, although aromatherapy has shown some efficacy, especially with single component essential oils, the effects of mixed herb aromatherapy have not been extensively studied. A pilot study is necessary not only to fill the existing knowledge gap but also to address practical issues such as the feasibility of the aromatherapy treatment among patients, which includes assessing recruitment, adherence, completion rates and safety assessment. In this study, we chose a combination of several traditional Chinese herbs for aromatherapy to explore its effects on PSD. The selected herbs include Farfarae Flos (6g), Lysimachia christinae Hance (6g), Mume Flos (6g), Sophora tonkinensis Gagnep (6g), Polygoni multiflori Caulis (6g), Ziziphi Spinosae Semen (6g), and Liquidambar formosana Hance (12g). The formulas of these herbs are derived from the summaries of experienced and renowned TCM practitioners, and involve the compounding and long-term experience of TCM. These herbs will be ground into a fragrant powder, and the aroma will be lit for patients to inhale. Each herb was selected based on TCM theory and for its unique pharmacological properties:

1. Farfarae Flos is one of the key components in our aromatherapy for treating PSD. It was selected for its diverse pharmacological properties, including anti-inflammatory, antioxidant, and neuroprotective effects. Research has shown that Farfarae Flos contains active compounds like flavonoids and sesquiterpenoids, which can inhibit inflammatory mediators such as TNF-α and IL-6, thereby reducing inflammation, neural damage, and depressive symptoms following a stroke. Additionally, its flavonoids exhibit strong antioxidant activities that mitigate oxidative stress, protecting neurons and promoting neural recovery (20).
2. Lysimachia christinae Hance(Jinqiancao) was chosen for its significant pharmacological properties, including anti-inflammatory, antioxidant, and neuroprotective effects. The herb’s main active compounds are flavonoids, phenolic acids, volatile oils, and polysaccharides. Research has shown that Jinqiancao can inhibit inflammatory mediators, reduce oxidative stress, and protect neurons from damage, supporting neural recovery and potentially alleviating depressive symptoms after a stroke. Flavonoids like quercetin and kaempferol present in Jinqiancao exhibit strong antioxidant activities, helping to mitigate oxidative stress and promote the recovery of neural functions (21).
3. Mume Flos was selected for its notable pharmacological properties, particularly its antidepressant, antioxidant, and neuroprotective effects. The primary active compounds in Mume Flos are flavonoids, phenylpropanoids, anthocyanins, volatile components, and organic acids. Research has demonstrated that the total flavonoids from Mume Flos can significantly decrease immobility time in forced swimming and tail suspension tests, indicating potent antidepressant effects. Additionally, these flavonoids help mitigate oxidative stress and protect neurons, which is crucial for post-stroke recovery. Mume Flos has also been shown to enhance serotonin levels, further supporting its use in alleviating depressive symptoms (22, 23).
4. Sophora tonkinensis Gagnep, included in our aromatherapy study, offers notable anti-inflammatory, antioxidant, and neuroprotective effects. Its flavonoids, such as quercetin and formononetin, inhibit inflammatory mediators, reduce oxidative stress, and protect neurons, which are crucial for alleviating depressive symptoms and supporting neural recovery post-stroke (24).
5. Caulis Polygoni Multiflori Caulis(Yejiaoteng), used in our aromatherapy study, is recognized for its significant sedative, anti-inflammatory, and neuroprotective properties. The primary active compounds include flavonoids, anthraquinones, and stilbenes. These compounds help reduce inflammatory cytokines, mitigate oxidative stress, and protect neurons. Additionally, Yejiaoteng enhances neurotransmitter regulation and improves sleep quality, which is crucial for alleviating depressive symptoms and supporting neural recovery in PSD patients (25, 26).
6. Ziziphi Spinosae Semen (Suanzaoren) is known for its significant antidepressant, anti-inflammatory, and neuroprotective properties. Its active compounds, such as jujubosides and flavonoids, modulate neurotransmitter levels, reduce inflammatory cytokines, and enhance brain-derived neurotrophic factor (BDNF) expression. These actions help alleviate depressive symptoms, protect neurons from damage, and support neural recovery, making Suanzaoren an effective ingredient in treating PSD (27, 28).
7. Liquidambar formosana Hance(Lulutong) is recognized for its significant anti-inflammatory, antioxidant, and neuroprotective properties. The primary active compounds are betulinic acid and gallic acid. These compounds help reduce capillary permeability, inhibit the secretion of inflammatory mediators, and protect neurons by maintaining calcium homeostasis and reducing oxidative stress. Additionally, Lulutong has shown potential in enhancing neurotransmitter regulation and alleviating depressive symptoms, making it an effective component in treating PSD (29). These pharmacological actions justify the inclusion of Lulutong in our PSD treatment approach.

In summary, these herbs work by inhibiting inflammatory mediators, reducing oxidative stress, protecting neurons, and regulating neurotransmitters. This multifaceted approach helps alleviate depressive symptoms, promote neural recovery, and improve overall emotional well-being, making our aromatherapy a potentially effective intervention for PSD.

Inhalation is chosen as the method of administration in this study because it allows the active compounds to enter the bloodstream directly through the lungs, potentially leading to fast therapeutic effects (30). Moreover, inhalation aromatherapy not only has physiological benefits but also psychological effects by stimulating the brain through olfactory pathways, thereby improving mood and emotional well-being (31). This non-invasive method is also convenient for patients, promoting better compliance and integration into their daily routines. The main mechanisms currently considered to be responsible for the improvement of PSD in patients by aromatherapy include: the olfactory pathway hypothesis, the limbic system and brainstem reticular formation hypothesis, the neurotransmitter hypothesis, the psychological hypothesis, and some TCM mechanisms (30–32). From the viewpoint of TCM mechanism, aromatic herbs have aromatic odor and volatile herbs (33), and aromatherapy is to extract and refine the effective aromatic components in plants under the guidance of TCM theory and give them to the patients through multiple routes of administration. TCM believes that aromatic herbs can enter the internal organs, which in TCM include the heart, liver, spleen, lungs, and kidneys. Each of these organs plays a crucial role in maintaining the body’s balance. For example, the liver is associated with regulating emotions and detoxification processes. The concept of “detoxifying” in TCM often refers to the process of removing toxins or harmful substances from the body, particularly from the liver. By detoxifying the liver, it is believed that the body’s balance is restored, which can alleviate symptoms such as depression and emotional disturbances. This is because the liver, in TCM, is thought to govern the flow of emotions and qi (vital energy) within the body. In TCM, qi flows through the body, maintaining health, and its disruption can lead to illness. Aromatherapy, by influencing the flow of qi, particularly lung qi, aims to balance the body’s internal energy and positively impact mental health. The lungs, linked to the nose, govern qi, making aromatic therapies effective. Furthermore, TCM theory states that aromatic herbs can harmonize internal organs by balancing their functions and interactions, thereby supporting overall health and well-being. In summary, aromatherapy may offer a unique approach to treating depression by acting on the olfactory system and following TCM theories on qi and the relationship between the lungs and the nose. Further scientific studies are needed to validate these effects and explore their specific biological mechanisms.

Aromatherapy, as an adjunctive treatment, is widely used to alleviate symptoms such as pain, anxiety, and depression, with its safety being well-documented in numerous studies. One systematic review highlights that inhalation aromatherapy shows significant efficacy in reducing stress and anxiety and is generally considered safe (34). Some research notes that while aromatherapy has potential benefits in managing pain, nausea, and preoperative anxiety, there are potential risks such as skin sensitivity, phototoxicity, and flammability. These risks need to be managed through clinical oversight and best practice models (35). Additionally, research discussing the effects of nasal-delivered aromatherapy on mood disorders provides less detailed discussion on safety issues, indicating a gap that needs to be addressed (36). While herbal powders have a long history in TCM, their safety as incense materials in modern clinical applications requires careful assessment. We will evaluate safety based on clinical monitoring. By recording any adverse reactions, we aim to provide evidence for the safe clinical use of herbal powder incense.

Evaluating the feasibility of implementing this innovative intervention in a clinical setting, including recruitment rates, intervention adherence, assessment completion, and safety assessment, is crucial for determining whether a full-scale RCT would be feasible and practical. Meanwhile, understanding the multifaceted impact of PSD is crucial, particularly considering the multiple symptoms of PSD and the effects documented in previous studies on anxiety, sleep, and other related symptoms. This study, therefore, assesses not only depressive symptoms but also cognitive function, sleep quality, and overall quality of life. These dimensions are integral to evaluating the comprehensive impact of PSD. Improvements in these areas are indicative of effective intervention and are vital for reducing the long-term burden on caregivers and healthcare systems.

For clinical screening of PSD, a variety of scales have been available for clinical use, including Hamilton Depression Rating Scale (HDRS), Beck Depression inventory (BDI), General Health Questionnaire (GDS), Hospital Anxiety and Depression Scale (HADS), and Patient Health Questionnaire-9 (PHQ-9). However, there are differences in the assessment range, sensitivity and specificity of each scale. Therefore, it has been suggested that at least two different assessment scales should be used in clinical screening to improve the accuracy of depressive symptoms (2).

Electroencephalogram (EEG) amplifies and records the spontaneous biopotentials of each functional brain region to understand the damage in the brain region that corresponds to the clinical symptoms. Zhang et al. (37) recorded resting-state closed-eye EEG signals from 21 patients with PSD, 22 post-stroke non-depression (PSND) patients, and 15 healthy individuals (controls, CONT), and assessed the complexity of EEG changes in PSD patients with Lempel-Ziv complexity (LZC). The results of this study showed that the neural complexity of the whole brain regions was lower in PSD than in PSND and CONT, but there were no significant differences between the different brain regions. Although there was no significant correlation between LZC and either the overall level of depression or the severity of the seven symptomatic differences in patients with PSD, there was a significant correlation between the HDRS and the LZC in patients with stroke, particularly in the frontal and temporal lobes. The LZC parameters used in the experiment for PSD identification had more than 85% specificity, sensitivity, and accuracy. However, since this is the only study reporting such findings, it cannot be concluded that LZC can be used to screen for PSD definitively. LZC can be considered as one of the alternative objective measurements, but more studies are required to ascertain its validity.

As a noninvasive, real-time and simple brain function imaging technique, functional near - infrared spectroscopy (fNIRS) has been widely used in neuroscience and clinical medicine in recent years. It monitors the metabolic activities and hemodynamic status of the brain in real time by measuring changes in blood oxygen levels in brain tissue, thus providing a visual reflection of brain functional activities. In the field of mental health, fNIRS technology has been used to diagnose and assess a wide range of psychiatric disorders, such as depression and anxiety disorders, and has demonstrated its potential advantages in early screening, assessing the severity of disorders, and guiding treatment protocols. The verbal fluency task (VFT) has been widely used to explore functional cognitive impairment and a variety of psychiatric disorders in fNIRS studies. Researchers have employed the VFT to test patients with depression, suggesting that this task paradigm can provide evidence-based support for the diagnosis of depression. For example, Matsuo et al. (38) investigated prefrontal cortical blood flow changes under a VFT and during hyperventilation in depressed patients, and found that prefrontal cortical activation was significantly lower under the VFT and the magnitude of prefrontal cortical blood flow changes during hyperventilation was smaller in depressed patients compared to normal subjects. Similarly, Zhang Fuxu et al. (39) discovered patients with major depressive disorder (MDD) have significantly lower hemodynamic response intensity in the prefrontal and bilateral temporal regions during the VFT compared to healthy controls, and that the integral value of the prefrontal region has diagnostic value in distinguishing MDD patient. Dong SY et al. (40) demonstrated that prefrontal functional connectivity assessed by fNIRS during word fluency testing can effectively differentiate between patients with MMD and healthy individuals, with network efficiency strongly correlated with depression severity.

Using VFT as an activation task, researchers investigated frontal lobe function in patients with PSD, and found that the fNIRS oxygen-hemoglobin score value was negatively correlated with 17-item Hamilton Rating Scale for Depression (HAMD-17) total score, and there was a significant difference between the oxygen-hemoglobin score value of the nondepressed group and that of the depressed group (41). These results suggest that fNIRS has potential in diagnosing PSD. Nevertheless, more studies are required to ascertain the validity, sensitivity, and specificity of fNIRS as a diagnostic tool for PSD. However, beyond the study by Koyanagi et al. (41), no other studies have replicated this finding. Additionally, there is still a lack of near-infrared research specifically focusing on this unique subset of depression, PSD. Therefore, while fNIRS shows promise as an objective measurement, it cannot yet be considered a definitive diagnostic tool for PSD. More studies are required to ascertain its validity, sensitivity, and specificity.

This study employs two comparators: Cognitive Behavioral Therapy (CBT) and non-active conventional treatment. CBT is included as a comparator due to its well-established efficacy and widespread acceptance as a standard treatment for various forms of depression, including PSD. The inclusion of a non-active conventional treatment group allows for the assessment of the absolute effects of aromatherapy, offering a baseline to observe changes attributable solely to the aromatherapy intervention without the influence of active therapeutic elements.

In summary, this pilot study aims to explore the feasibility and potential benefits of mixed herb aromatherapy as an innovative and accessible treatment option for PSD. Aromatherapy, often less resource-intensive than CBT, could offer a complementary approach, particularly beneficial in settings where traditional therapies are limited. This pilot phase will assess the practicality of integrating aromatherapy into existing treatment protocols for PSD and evaluate its preliminary effects on patient outcomes such as mood improvement, anxiety reduction, and overall quality of life. The insights gained from this initial exploration will help refine the intervention and prepare for a more comprehensive randomized controlled trial. The study will also assess anxiety, cognitive function, sleep quality, and quality of life. These measures are important because post-stroke depression can impact multiple aspects of a patient’s life, and improvements in these areas can contribute to overall recovery and well-being.

## 2 Objectives

The objectives of this study are:

1. To assess the feasibility of implementing the mixed herb aromatherapy intervention in a clinical setting. Key metrics include recruitment rates, adherence rates, completion rates and safety assessment.
2. To gather preliminary data on the efficacy of mixed herb aromatherapy on PSD, including symptom relief and improvements in the quality of life of the patients.
3. To compare the preliminary effects of aromatherapy versus routine treatment and CBT for PSD patients, focusing on differences in symptom recovery and sustainability of treatment effects.
4. To explore the impact of aromatherapy on brain function assessments (EEG and fNIRS) and its correlation with the recovery of patients suffering from PSD. This study will utilize EEG and fNIRS to assess the impact of aromatherapy on brain function in PSD patients and investigate the correlation between these brain function changes and the recovery progress of the patients.

## 3 Trial design

A single-blind, pilot randomized controlled trial will be conducted to evaluate the preliminary efficacy and feasibility of mixed herb aromatherapy compared to standard care and CBT. This study will enroll ninety-nine post-stroke patients with PSD at the Second Rehabilitation Hospital of Shanghai. Prior to initiating rehabilitation, demographic details of all participants, including name, gender, age, education level, and medical history, will be collected. A centralized, web-based service provided by Research Randomizer (https://www.randomizer.org/) will be utilized to generate a randomization sequence, ensuring an even distribution of patients between the three groups: the non-active control group (receiving standard care), the aromatherapy group (undergoing daily aromatherapy inhalation), and the CBT group (receiving CBT sessions twice weekly) in a 1:1:1 ratio. The intervention will last for four weeks.

Primary feasibility outcomes will include recruitment rates, adherence to the intervention and completion rates. Recruitment rates will be monitored and documented weekly. Adherence will be tracked through participant logs, ensuring that each session is recorded. Completion rates will be calculated at the study’s conclusion.

**Figure 1:**
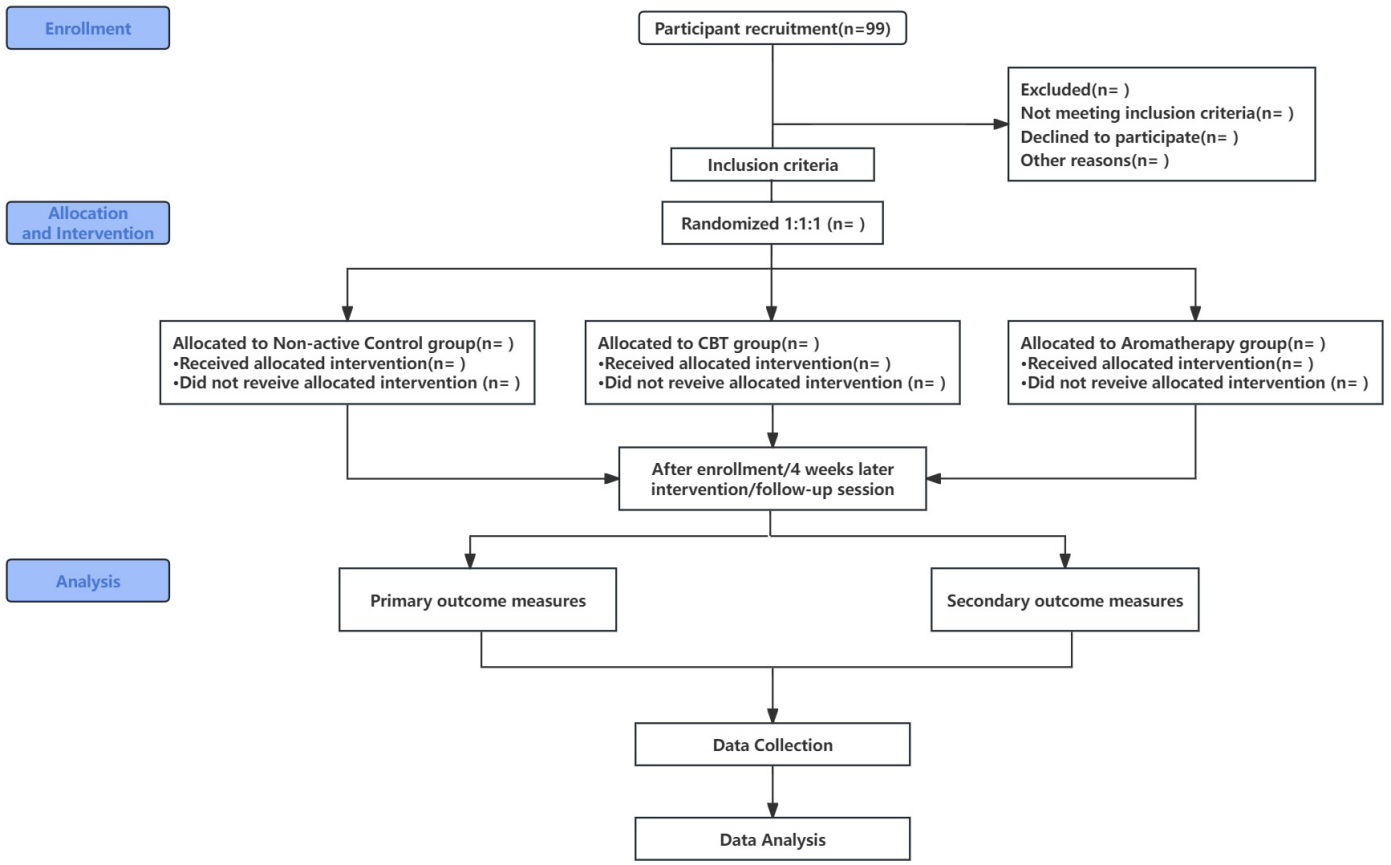
Clinical Trial Flow Chart.

Evaluation of intervention effectiveness will be conducted at three time points: before the intervention (baseline), immediately after the intervention (four weeks), and one month post-intervention, to assess the effectiveness of the treatments (see **Figure 1** for details).

## 4 Methods: participants, interventions and outcomes

### 4.1 Study setting

This study will be conducted at the Second Rehabilitation Hospital of Shanghai, China. This hospital is a comprehensive rehabilitation institution offering multidisciplinary rehabilitation treatment services, equipped with the facilities and professional staff required for the execution of this study. Located in the central area of Shanghai, the hospital is easily accessible for participants. Data will be collected in China.

#### 4.1.1 Eligibility criteria

##### 4.1.1.1 Inclusion criteria

- Diagnosed by cranial CT or MRI and meeting the diagnostic criteria for stroke as revised by the 4th Cerebrovascular Disease Conference.

- Meet the diagnostic criteria for depression in Chinese Classification and Diagnostic Criteria for Mental Disorders CCMD-3.

- Age 30~80 years old, gender is not limited.

- PHQ-9 score ≥5.

- No severe cognitive impairment.

- Those with no history of depression before stroke.

- Normal sense of smell.

- Informed consent and voluntary enrollment in the study.

##### 4.1.1.2 Exclusion criteria

- Stroke patients with unstable condition.

- Patients with serious bone, joint, and muscle disorders; liver or kidney diseases; other serious life-threatening diseases (e.g., advanced cancer, severe cardiovascular diseases, severe respiratory diseases, end-stage liver or kidney diseases); and serious mental illnesses such as schizophrenia or severe bipolar disorder.

- Patients with severe speech, visual and hearing impairments.

- Patients who have received aromatherapy within one month.

- Patients with asthma, history of respiratory diseases such as allergy to flowers, plants and essential oils.

- Patients with scalp trauma, skin breakdown, redness, inflammation, or soft tissue damage that would interfere with the examination.

- Patients with a history of seizures.

- Patients undergoing other clinical trials.

##### 4.1.1.3 Withdrawal or dropout criteria

- Participants with incomplete or false information.

- Participants with adverse events.

- Participants who are not suitable for continuing treatment due to other systemic diseases for various reasons.

- Participants who voluntarily proposed to withdraw from the study.

#### 4.1.2 Who will take informed consent?

In this clinical trial, the responsibility for ensuring that potential participants understand and agree to participate will be assumed by qualified and professionally trained members of the research team, which primarily includes the Principal Investigator in charge of the project and/or the Clinical Research Coordinator. These professionals have completed comprehensive training in ethical research practices, including Good Clinical Practice (GCP) certification, to ensure that they have the ability to communicate with potential participants in a clear, detailed and non-coercive manner. The informed consent process will be conducted through a series of detailed and transparent steps to ensure that each potential participant fully understands the objectives, process, possible risks and benefits of the study, as well as their rights and obligations. This process includes providing a comprehensive Participant Information Sheet (PIS) that details the study’s purpose, procedures, potential risks and benefits, confidentiality and data security measures, and the voluntary nature of participation, including contact information for the research team. Eligible patients will be given at least 48 hours to review the PIS, ask any questions, and discuss their potential participation with family, friends, or healthcare providers if they wish. This period is designed to ensure that patients do not feel rushed and can thoroughly consider all aspects of the study before providing their consent. This process involves a one-on-one meeting with the participant, during which the researcher will brief them in detail about the study and answer any questions, ensuring that each participant fully understands the study’s objectives, procedures, possible risks and benefits, and their rights and obligations. Written consent will only be collected when it is confirmed that the participant has been fully informed of all relevant information and is voluntarily agreeing to take part in the study. Patients included in this study will not have severe cognitive impairment, but for those participants with limited mobility, consent will be sought from their legal guardian or authorized agent, ensuring that the agent fully understands the research and consents on behalf of the participant. By implementing these measures, we are committed to upholding the rights of participants and ensuring that the research complies with ethical and legal standards.

#### 4.1.3 Additional consent provisions for collection and use of participant data and biological specimens

This study does not plan to collect or use participants’ data and biospecimens for any ancillary research. Therefore, participants will not be asked to provide additional consent for this purpose. All data and specimens collected will be used only for the primary research purpose of this study and in strict compliance with relevant ethical and privacy protection standards.

### 4.2 Interventions

#### 4.2.1 Explanation for the choice of comparators

In this study, we aim to evaluate the effectiveness of CBT and Aromatherapy Inhalation interventions in the rehabilitation of PSD. To ensure the accuracy and reliability of our results, we have meticulously chosen the following comparators:

##### 4.2.1.1 Conventional rehabilitation treatment

Serving as the baseline comparison, all participants will receive standard rehabilitation treatments, including physical therapy, occupational therapy, acupuncture and medication management, to meet basic rehabilitation needs. This control group allows us to assess the additional benefits of CBT and Aromatherapy Inhalation interventions on top of standard care.

##### 4.2.1.2 Cognitive behavioral therapy intervention

CBT is a psychosocial intervention that operates on altering patients’ cognitive and behavioral patterns to improve emotional states and functionality. The reason for selecting CBT as one of our comparators is its well-documented effectiveness in treating depression, including PSD. By including CBT as an intervention, we aim to evaluate its specific utility in the domain of stroke rehabilitation.

Through comparing these interventions, our study hopes to reveal the specific impacts and benefits of CBT and Aromatherapy Inhalation interventions on the rehabilitation process of patients with PSD, beyond conventional care. Moreover, the selection of comparators is based on the feasibility of interventions, patient acceptability, and the evidence base from previous research, aiming to provide empirical support for the comprehensive treatment of PSD.

#### 4.2.2 Intervention description

##### 4.2.2.1 Non-active control group

All participants receive routine standardized conventional rehabilitation treatment, with interventions delivered by the standardized rehabilitation therapy components of the Second Rehabilitation Hospital of Shanghai. Five times a week for a total of four weeks. This specialized program will be delivered by a team of 6 certified professionals, including 2 physical therapists (PTs), 2 occupational therapists (OTs), and 2 acupuncture physicians, ensuring a holistic approach to patient care. To further enhance the quality and consistency of the treatment, all therapists will receive standardized training prior to the research. This enhanced approach aims to deliver a holistic and standardized treatment experience, blending physical therapy (exercise therapy, balance training, and joint mobilization), occupational therapy (including homework training, hand training, and cognitive dysfunction training), acupuncture therapy, and health education.

##### 4.2.2.2 CBT group

The CBT group will participate in a comprehensive rehabilitation regimen that integrates conventional treatments with Cognitive Behavioral Therapy (CBT), administered by trained psychotherapists. The inclusion of CBT aims to improve mental health and coping strategies, ensuring a comprehensive treatment protocol that addresses both the physical and psychological aspects of recovery for the control group. Participants will receive standard rehabilitation treatments in addition to CBT sessions conducted twice weekly, each lasting 30 minutes, over four weeks. The interventions include:

###### 4.2.2.2.1 Emotional journaling

At the start of each session, patients will report on emotional changes experienced since their last session, detailing specific emotions, triggers, and duration.

###### 4.2.2.2.2 Introduction to cognitive restructuring

Discussions will focus on identifying and challenging negative thought patterns and their impact on emotions, guiding patients towards a more positive mindset.

###### 4.2.2.2.3 Behavioral activation strategies

Patients will be encouraged to engage in daily activities that generate positive emotional feedback, discussing challenges and coping strategies encountered during these activities.

###### 4.2.2.2.4 Skill practice and application

Through case studies and role-playing, patients will strengthen their ability to apply CBT techniques, better equipping them to handle everyday challenges.

###### 4.2.2.2.5 Summary and goal setting

Each session will conclude with a summary of the session’s content and the setting of goals and tasks for patients to complete before the next session.

##### 4.2.2.3 Aromatherapy group

Aromatherapy group is based on conventional rehabilitation treatment, patients are given aromatic inhalation treatment for 4 weeks, once a day, 30 minutes each time. The aromatherapy intervention will be administered by physicians from the Traditional Chinese Medicine (TCM) Rehabilitation Department, who have received specialized training in herbal powder aromatherapy. The aromatherapy will be conducted daily for 30 minutes, and participants will need to travel to the designated area within the hospital every day for this 30-minute intervention. Since all patients are inpatients, they will simply need to coordinate the timing with the hospital schedule. Selected herbs (Farfarae Flos 6g, Lysimachia christinae Hance 6g, Mume Flos 6g, Sophora tonkinensis Gagnep 6g, Polygoni multiflori Caulis 6g, Ziziphi Spinosae Semen 6g, Liquidambar formosana Hance 12g) will be ground into fragrant powder, and the aroma will be lit for patients to inhale in a 15 m² treatment room. Ensure that the diffused aromatic odor can fully fill the space but not overpowering.

In addressing safety concerns, the study excludes individuals with respiratory conditions like asthma or allergies, to avoid any potential exacerbation of symptoms. The therapy sessions will be held in a treatment room within the hospital that is equipped with a ventilation device. This ensures effective dispersal of the aromatic smoke and maintains the indoor air quality. The ventilation device provides continuous air circulation to keep the smoke concentration at a safe level. Additionally, each therapy session will be closely monitored by medical professionals who are ready to manage any adverse reactions immediately. These measures ensure the safety of participants while adhering to the ethical standards of the research.

All interventions will be conducted at the rehabilitation hospital. Since all patients are inpatients, they will only need to coordinate with the staff to schedule and participate in the intervention sessions, ensuring convenience and consistency. All groups will receive the same patient-centered and standardized conventional treatment protocol, which is uniformly administered across all participants to ensure consistency. However, the CBT group and the aromatherapy group will receive 4 weeks of corresponding treatment in addition to the conventional treatment.

#### 4.2.3 Criteria for discontinuing or modifying allocated interventions

For patients with PSD who received the aromatherapy inhalation intervention, if a serious adverse reaction (e.g., an allergic reaction) occurs during the treatment, the research team will immediately stop the treatment for that participant and carry out the necessary medical interventions. At the same time, if the participant voluntarily requests to stop the treatment, we will record his/her request and respect his/her decision. Participants’ depressive symptoms will be regularly assessed in the study and adjustments to the treatment regimen will be considered if significant improvement or worsening is observed. All these adjustments will be made under the guidance of the research ethics committee to ensure that the safety and rights of the participants are protected.

#### 4.2.4 Strategies to improve adherence to interventions

Participant compliance will be ensured in the following three points. (1) When recruiting subjects and signing the informed consent form, the researchers will explain in detail to the patients about the needs of the subjects during the observation period and establish a good relationship with the subjects to gain more trust. (2) During the trial, researchers will provide comprehensive education and guidance on disease management and preventive measures. This includes teaching patients about the nature of their condition, the importance of adherence to the treatment protocol, and strategies to prevent complications. By enhancing patients’ understanding and involvement in their own care, we aim to foster better compliance and active participation. (3) All participants in the intervention and control groups need to record participation in the intervention on a schedule. Ensure that the number of weekly interventions is achieved.

#### 4.2.5 Relevant concomitant care permitted or prohibited during the trial

It is stated in the inclusion criteria that participants can not participate in other clinical trials and that there will be no treatment other than the usual treatments and interventions.

#### 4.2.6 Provisions for post-trial care

N/a

### 4.3 Outcomes

The primary objective of this pilot RCT is to evaluate the feasibility of implementing mixed herb aromatherapy for treating post-stroke depression, and to determine if a full-scale RCT would be feasible. Feasibility outcomes, which will be the primary outcomes, include recruitment rates, adherence rates, and completion rates. Secondary outcomes will assess changes in depression, sleep quality, cognitive level, quality of life, and cerebral cortex activity in PSD participants after different interventions, evaluated using five rating scales, EEG testing, and fNIRS. Specifically, the details are as follows.

#### 4.3.1 Primary outcome measures

1. Recruitment rates: Recruitment rates will be calculated as the proportion of successfully recruited eligible participants within the specified period, documented weekly, and reported as a percentage of the total planned recruitment. A recruitment rate of at least 70% will be considered acceptable.

2. Intervention adherence: Intervention adherence will measure the proportion of participants who complete the intervention as planned. This will be tracked using participant logs that record the completion of each aromatherapy session, CBT session, and conventional treatment session. Adherence to each component will be reported weekly. An adherence rate of at least 80% will be considered acceptable. Additionally, feasibility outcomes will include the proportion of participants who complete all assessments as scheduled, assessing the practicality of integrating aromatherapy with existing treatments.

3. Assessment completion: Assessment completion will assess the proportion of participants who complete all scheduled assessments and follow-ups. This will be calculated and reported at the end of the intervention. A completion rate of at least 85% will be considered acceptable.

5. Safety Assessment: Safety will be evaluated through subjective and objective measures. Participants will report any discomfort or adverse reactions, and vital signs (heart rate, respiratory rate, blood pressure) will be monitored before and after each session. An adverse event log will document all incidents and management actions. Adverse reactions will be categorized as mild, moderate, or severe.

- Moderate: Noticeable symptoms that may interfere with daily activities but are not life-threatening, requiring medical intervention or short-term observation.

- Severe: Symptoms significantly impacting daily life, potentially life-threatening, requiring intensive medical intervention such as hospitalization or emergency medication.

An acceptable safety threshold is set at no more than 10% of participants experiencing moderate to severe adverse reactions.

#### 4.3.2 Secondary outcome measures

1. Patient Health Questionnaire-9 (PHQ-9): The Chinese version of PHQ-9 has been translated and validated, demonstrating good validity and reliability for assessing depression severity among Chinese populations (42). It consists of nine items representing various aspects of depressive symptoms with high reliability and easy to understand. Each item is rated on a four-point scale, with the patient choosing the most compatible answer based on how they felt over a two-week period. 0: no problem at all; 1: a few days; 2: most of the time; 3: every day. The total score ranges from 0 to 27, with higher scores indicating more severe depressive symptoms. Score 0 to 4: no or minimal depressive symptoms; 5 to 9: mild depression; 10 to 14: moderate depression; 15 to 19: moderately severe depression; 20 to 27: severe depression.
2. Hospital Anxiety and Depression Scale: We will use the translated and validated Chinese version of the HADS. This version has demonstrated good validity and reliability in studies involving Chinese populations (43). The self-report scale is widely used to assess patients’ levels of anxiety and depression in healthcare settings. The scale was developed by Zigmond and Snaith in 1983 and is primarily designed to be used as a quick and easy measure of a patient’s psychological state under the guidance of a non-psychological professional (44). The HADS consists of 14 items, of which 7 items are used to assess depression (Items #2, 4, 6, 8, 10, 12, and 14 items) and 7 items of the scale are used to assess anxiety (Items #1, 3, 5, 7, 9, 11, and 13). Each item has a 4-point scale, with each item scored on a scale ranging from 0 to 3, and the total score ranging from 0 to 21.
3. Mini-Mental State Examination (MMSE): We will use the translated and validated Chinese version of the MMSE. This version has been widely applied in various studies and has proven effective and reliable for assessing cognitive function in Chinese populations (45). The MMSE is a simple instrument widely used for the assessment of cognitive functioning and consists of 30 items with a total score of 30 points. It includes attention and calculation ability (5 points), memory (3 points), orientation (10 points), language (8 points), and executive function (4 points). 0-9: moderate cognitive disorder; 10-17: mild cognitive disorder; 18-23: mild cognitive impairment; and 24-30: normal cognitive level.
4. Pittsburgh sleep quality index (PSQI): We will use the Chinese translated version of the PSQI. The translated version has been validated and shown to be effective and reliable in assessing sleep quality among Chinese populations (46). The main purpose of the PSQI is to comprehensively assess the quality of an individual’s sleep, including sleep duration, difficulty falling asleep, sleep effect, sleep depth, number of sleep awakenings, and sleep medication use (47). It contains 7 main subscales that assess different aspects of sleep: subjective sleep quality, difficulty falling asleep, sleep duration, sleep effect, sleep depth, use of medications, and daytime dysfunction. Each subscale has a score range of 0-3 and a total score of 21, with higher scores representing poorer sleep quality. a total PSQI score of >5 is usually considered poor sleep quality.
5. Stroke Specific Quality of Life Scale (SS-QOL): We will use the translated and validated Chinese version of the SS-QOL. This version has demonstrated good validity and reliability for assessing the quality of life in Chinese stroke patients (48). The scale includes 12 dimensions of energy, family role, language, activity, mood, personality, self-care, social role, thinking, upper limb function, vision, and work/productivity, with each entry on a 5-point scale (1 to 5). The total score ranged from 49 to 245, with higher scores indicating better quality of life.
6. A 32-channel wireless multichannel EEG acquisition and analysis system from Xi’an ZhenTec Intelligence Technology, ZhenTec NT1, will be used for brain EEG data acquisition. Saline electrodes will be used and placed according to the international standard lead 10-20 electrode system position. The sampling rate will be 1000 Hz and electrode impedance will be kept below 10 kΩ. Resting state EEG will be recorded from the patients before and after four weeks of intervention. Resting-state EEG: EEG data will be recorded for 5 minutes in a quiet state. LZC complexity nonlinear feature extraction will be performed for the full frequency band (0.6-46Hz), δ (1-3.5Hz), θ (4-7Hz), α (8-13Hz), and β (13-30Hz) frequency bands of the resting-state EEG, respectively, to calculate the LZC values.
7. A 64-channel fNIRS device (BS-2000L, Wuhan Znion Technology Co., Ltd., China) will be used to collect information on changes in oxygenated hemoglobin (HbO) and deoxyhemoglobin (HbR) concentrations in the prefrontal lobe and part of the temporal lobe of the subjects during the VFT task. The VFT consists of the following components: (1)A 30-second pre-task baseline period to establish a resting state, during which participants will be asked to repeat the sequence “12345” to ensure consistent starting conditions. (2)A 60-second task period divided into three 20-second segments, during which participants are asked to generate as many words as possible starting with given syllables (e.g., “ba,” “da,” “pa”). (3)A 70-second post-task baseline period during which participants will again repeat the sequence “12345” until the end of the assessment at 170 seconds. This task evaluates regional brain activity and forebrain functional network connectivity. Data preprocessing includes artifact removal and baseline correction using the moving average method. HbO and HbR integral values will be calculated by averaging data from all channels, representing the hemodynamic response during the task period.

### 4.4 Participant timeline

The schedule for the study is shown in **Table 1**.

**Table 1:** Participant timeline.

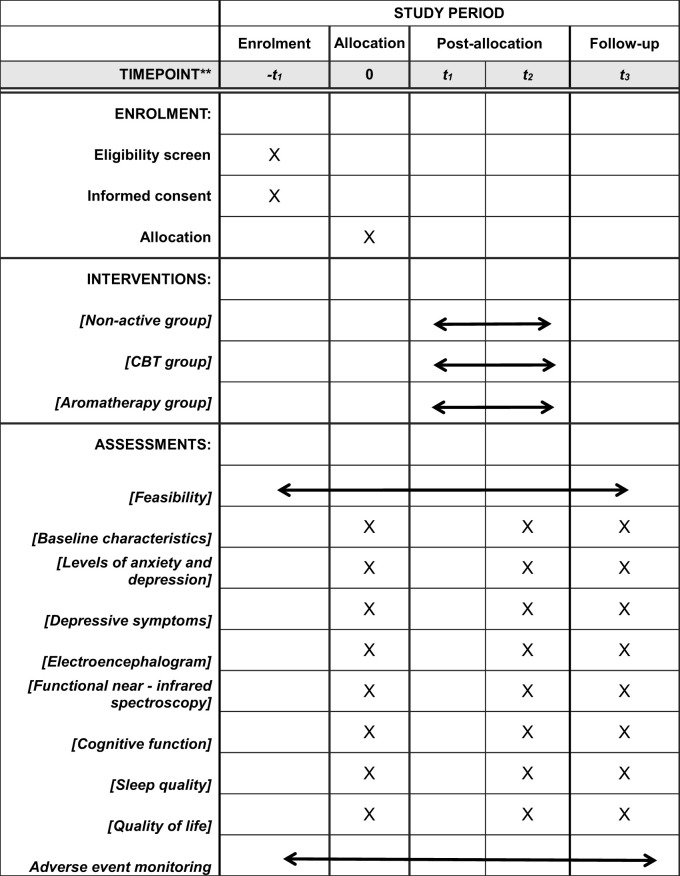	

### 4.5 Sample size and feasibility criteria

Since this study employs a novel mixed herbal aromatherapy intervention, there is no prior research directly comparable to reference. As this is a pilot RCT, the focus is on assessing the feasibility of the intervention rather than hypothesis testing. Therefore, the sample size estimation is not based on power analysis but according to rule of thumb.

Based on the guidance from Billingham et al. (49), a sample size of approximately 30 participants per group is considered appropriate for a pilot study. This sample size is justified by the need to gather preliminary data on feasibility outcomes, such as recruitment rates, adherence rates, and completion rates, and to ensure sufficient variability and precision in the estimates.

Taking into account 10% potential dropout rates, we will aim to recruit approximately 33 participants per group to ensure we have enough data for the feasibility analysis. This will result in a total sample size of approximately 99 participants across the three groups.

The predefined criteria for feasibility outcomes are:

- Recruitment rate of at least 70%: This is considered a reasonable and achievable target for pilot studies to ensure adequate participant recruitment.

- Adherence rate of at least 80%: This reflects a high level of participant engagement and compliance with the intervention protocol, which is crucial for obtaining reliable data.

- Completion rate of at least 85%: This indicates that the majority of participants complete the study, ensuring data integrity and minimizing the impact of dropouts on the study results.

These criteria will help determine the feasibility of conducting a full-scale RCT.

### 4.6 Recruitment

All participants will be recruited at the Second Rehabilitation Hospital of Shanghai, where research advertisements will be posted to attract participants. To ensure the quality of recruitment, the chief investigator will oversee the review of medical records. The recruitment process will be conducted by two dedicated researchers, who will meticulously document the participants’ medical histories, both past and present.

## 5 Assignment of interventions: allocation

### 5.1 Sequence generation

Participants will be randomized in a 1:1:1 ratio to control group, CBT group or aromatherapy group. Due to the unique characteristics of each intervention, complete blinding of participants and therapists is not possible. However, measures will be taken to minimize bias, including withholding specific hypotheses about the interventions from participants. Data collection will be performed by investigators blinded to group assignments. Furthermore, data analysis will occur under blinded conditions, with assignments coded and concealed until the conclusion of the analysis.

### 5.2 Concealment mechanism

The allocation sequence will be implemented through a centralized, web-based service provided by Research Randomizer (https://www.randomizer.org/). An independent team member, uninvolved in participant enrollment or intervention assignment, will conduct the randomization to ensure the sequence’s concealment. Group assignments will then be relayed to the team administering the interventions, safeguarding against any prior knowledge of participant assignments by the enrolment and intervention teams. This strategy guarantees the sequence’s concealment until assignment, maintaining allocation concealment. Additionally, researchers collecting data will be blinded to assignments, reducing bias. The coding and concealment of group assignments will be maintained until the analysis phase is completed.

### 5.3 Implementation

An independent statistician, separate from the participant enrollment and intervention administration processes, will generate the allocation sequence using Research Randomizer software (https://www.randomizer.org/), ensuring a balanced 1:1:1allocation to control group, CBT group or aromatherapy group. Trained research staff will manage participant enrollment, screening for eligibility without access to the allocation sequence, thus remaining uninformed of group assignments. Following consent, a distinct research team member will access the allocation via a secure system to assign participants to interventions, communicating these assignments to the intervention administrator responsible for treatment delivery. This administrator will be excluded from the enrolment and outcome assessment processes to avoid potential biases.

CBT will be delivered by the hospital unit’s psychotherapists. The aromatherapy intervention, utilizing traditional Chinese herbal powders, will be administered by physicians from the TCM Rehabilitation Department. These physicians have received specialized training in herbal powder aromatherapy, which includes:

1. Fundamentals of Aromatherapy: Understanding the principles and therapeutic benefits of aromatherapy using Chinese herbal powders.
2. Application Techniques: Hands-on training on the proper preparation and application of herbal powders for aromatherapy.
3. Safety Protocols: Comprehensive instruction on safety measures to prevent adverse reactions and ensure patient safety during the treatment.
4. Patient Interaction: Techniques for effectively communicating with patients to enhance their comfort and cooperation during the therapy.

The training program is conducted by senior TCM specialists and certified aromatherapists, ensuring that the physicians are proficient in delivering the intervention effectively and safely. During the aromatherapy sessions, these trained physicians will accompany patients throughout the treatment to ensure proper and safe application.

## 6 Assignment of interventions: blinding

### 6.1 Who will be blinded?

Specifically, researchers responsible for evaluating the outcomes of the study will not have knowledge of the participants’ group assignments, ensuring that assessments are conducted objectively and impartially. Similarly, the researchers who perform data analysis will be kept unaware of the group assignments. The data will be encoded and kept concealed throughout the analysis phase until its conclusion. Through these measures, we aim to reduce potential bias by ensuring that those assessing outcomes and analyzing data do not have preconceived notions about the participants’ intervention groups.

### 6.2 Procedure for unblinding if needed

Unblinding in this study is permitted under specific circumstances that warrant such action. These circumstances include emergency medical needs, where a participant experiences a serious adverse event or requires urgent medical intervention, and the knowledge of the group assignment is crucial for their treatment. Additionally, after the study concludes, requests for unblinding based on valid medical reasons by the participant or their healthcare provider will be considered. The procedure for unblinding involves a written request by the principal investigator or a member of the clinical oversight team. The clinical review panel is responsible for reviewing all unblinding requests to assess if the conditions for unblinding are met. Upon approval, the research team will follow a predefined protocol to disclose the participant’s group assignment safely. This protocol is designed to ensure that the unblinding process does not adversely affect the study’s integrity or the participant’s welfare, adhering to ethical guidelines and preserving the scientific value of the research.

## 7 Data collection and management

### 7.1 Plans for assessment and collection of outcomes

All questionnaires and assessment tools that will be used in this study have been validated for reliability and validity in previous studies. Cerebral cortex conditions will be acquired with standardized fNIRS and EEG equipment. All assessors will receive specialized training to ensure consistency and accuracy of assessments. Data entry will be performed using an electronic data capture (EDC) system and regular data quality audits will be implemented.

### 7.2 Plans to promote participant retention and complete follow-up

The follow-up of this study is scheduled 4 weeks after the end of the intervention. Regular contact will be maintained with participants by phone, email, or text message to provide study progress updates, answer participant questions, and remind of upcoming follow-up visits.

### 7.3 Data management

All data will be entered through the EDC system and a Unicode scheme will be used to maintain anonymity. Data will be stored on a password-protected server, accessible only to authorized researchers. Regular data backups will be performed to prevent data loss or corruption.

### 7.4 Confidentiality

All personal information, including name, gender, age, educational level, and medical history, will initially be collected. However, to ensure participant confidentiality, all identifying information will be de-identified after collection. This process involves replacing personal identifiers with unique codes. Only a limited number of authorized research team members will have access to the key linking these codes to the actual identifiers, which will be stored separately and securely. We will not share any personal information with third parties without obtaining explicit consent from the participant. All members of the research team will be required to sign a confidentiality agreement to uphold privacy standards. Throughout the study and after its conclusion, all personal information will be securely stored on a server accessible only to authorized researchers. We will implement appropriate technical and organizational measures to safeguard the data against unauthorized access, disclosure, or loss.

### 7.5 Plans for collection, laboratory evaluation and storage of biological specimens for genetic or molecular analysis in this trial/future use

This clinical trial does not involve the collection, laboratory evaluation, or storage of biological specimens (e.g., blood, tissue, etc.) for genetic or molecular analysis.

## 8 Statistical methods

### 8.1 Statistical methods for primary and secondary outcomes

Data analysis will be performed using SPSS 26.0. All data will be described by mean ± standard deviation (X ± S), with P < 0.05 considered statistically significant. Descriptive statistics will summarize the data. Confidence intervals and effect sizes will be reported where applicable, focusing on feasibility as the primary outcome and clinical measures as secondary outcomes.

#### 8.1.1 Primary outcome analysis: feasibility metrics

Recruitment, adherence, and completion rates will be the focus, analyzed descriptively to provide insights into the practical implementation and scalability of the intervention. Each metric will be reported as a percentage:

- Recruitment rate: Proportion of eligible participants who were successfully recruited.

- Intervention adherence: Proportion of participants who adhered to the intervention throughout the study.

- Assessment completion: Proportion of participants who completed all assessments and follow-ups.

- Safety assessment: Safety will be evaluated through subjective and objective measures, including monitoring vital signs and documenting adverse reactions.

#### 8.1.2 Secondary outcome analysis: clinical measures

- Measures such as PHQ-9, HADS, MMSE, PSQI, and SS-QOL will be summarized at each time point using descriptive statistics to observe potential effects and trends. Given the limited sample size, inferential analysis will be avoided. Instead, the focus will be on descriptive statistics and comparison with minimal important changes (MIC) to assess the clinical relevance of observed changes.

- To compare the efficacy of aromatherapy versus CBT, non-inferiority tests will be conducted to determine if the efficacy of aromatherapy is not significantly worse than that of CBT. The non-inferiority margin will be defined *a priori*, typically set at a 10% margin, indicating that aromatherapy is considered non-inferior if the difference in improvement between CBT and aromatherapy falls within this margin. If assumptions for parametric tests are violated, descriptive statistics will be used to summarize the data. This analysis will highlight the potential of aromatherapy as an effective complementary therapy.

- EEG data will be processed using a bandpass filter (0.6-46 Hz) and artifacts will be removed using Independent Component Analysis (ICA). A minimum of 4 minutes of artifact-free data will be selected for further analysis. The Lempel-Ziv Complexity (LZC) values for the full frequency band (0.6-46 Hz) and specific bands: δ (1-3.5 Hz), θ (4-7 Hz), α (8-13 Hz), and β (13-30 Hz) will be summarized at baseline, post-intervention, and follow-up within each group. Changes in LZC values over time and between groups will be explored descriptively, and the relationship between changes in LZC values and improvements in PHQ-9 scores will be examined using Pearson correlation to identify potential correlations between EEG biomarkers and clinical measures of depression. This analysis will help to understand if EEG changes can be predictive of or associated with improvements in depressive symptoms, providing preliminary insights into the neurological impacts of the interventions.

- The fNIRS data will collect information on changes in oxygenated hemoglobin (HbO) and deoxygenated hemoglobin (HbR) concentrations in the prefrontal lobe and part of the temporal lobe during the Verbal Fluency Task (VFT). The VFT will consist of a 30-second pre-task baseline, a 60-second task period, and a 70-second post-task baseline to assess changes in regional brain activity and forebrain functional network connectivity. The fNIRS data will be preprocessed to remove artifacts, with baseline correction performed using the moving average method. The last 10 seconds of the pre-task period and the first 55 seconds of the post-task period will be used for baseline correction. Descriptive statistics will summarize the integral values of HbO and HbR across different groups and time points. Changes in HbO and HbR values over time and between groups will be explored descriptively, and the relationship between HbO values and PHQ-9 scores will be examined using Spearman’s rank correlation.

### 8.2 Interim analyses

It is not expected that data will be analyzed for all participants until the end of the study.

### 8.3 Methods for additional analyses (e.g. subgroup analyses)

N/a

### 8.4 Methods in analysis to handle protocol non-adherence and any statistical methods to handle missing data

Missing data and any data collected that is not in compliance with the protocol will be removed from the data analysis.

### 8.5 Plans to give access to the full protocol, participant level-data and statistical code

Study datasets are available from the corresponding author upon reasonable request.

## 9 Oversight and monitoring

### 9.1 Composition of the coordinating center and trial steering committee

The Second Rehabilitation Hospital of Shanghai, will be the trial coordinating center. The Steering Committee (chaired by JH) is responsible for the final protocol, recruiting participants, assessing and reporting any serious unexpected adverse events, reviewing the progress of the study, and ensuring smooth running of the study. The Trial Manager (YL) is responsible for the trial’s master file, obtaining approval from the Ethics Committee, coordinating audits with the Ethics Committee, managing enrollment and allocation, releasing official changes of the clinical trial protocol to relevant entities and trial participants, scheduling participant assessments, and addressing any questions that participants have. XZ and YW will perform the intervention. Two researchers (ZH and HX) will perform data collection. YL will be responsible for data analysis.

### 9.2 Composition of the data monitoring committee, its role and reporting structure

Given that the Hospital Ethics Committee and Study Steering Committee have determined that the trial is relatively low risk, there will be no formal data monitoring committee. Data will be managed by a designated data manager. The Trial Manager (YL) will oversee data recording and resolve any issues regarding cloud storage, data management and data access. YL will frequently check and ensure that data collection is accurate, reliable and in accordance with protocol requirements.

### 9.3 Adverse event reporting and harms

The study is not expected to result in serious adverse reactions and the risk assessment will be monitored by clinicians. In the event of an adverse reaction caused by aromatic sniffing, we will promptly treat or withdraw the participant from the study. In the meantime, we will record the details in the case report form.

### 9.4 Frequency and plans for auditing trial conduct

The Hospital Ethics Committee conducts audits every six months to check progress and ensure compliance with the established protocol. Prior to the start of the trial, the Principal Investigator will meet with the study team on a weekly basis to ensure that all procedures are in line with the protocol standards. During the data collection phase of the trial, the team will meet monthly and the Principal Investigator (JH) and Trial Manager (YL) will be in daily contact to ensure compliance with the protocol.

### 9.5 Plans for communicating important protocol amendments to relevant parties (e.g. trial participants, ethical committees)

All revisions will initially be discussed internally by the steering committee to reach a consensus before being submitted to the hospital’s ethics committee and the research department. Upon approval, a formal document detailing the modifications to the protocol will be shared with all relevant parties, with an electronic version available upon request. Any deviations from the protocol will be documented by the trial manager and signed off by the principal investigator. This document will then be stored alongside all other trial documentation. Following approval from the steering committee, research department, and ethics committee, the trial manager will make the necessary updates to the clinical trial application.

## 10 Dissemination plans

A summary of the RCT outcomes will be compiled and emailed to participants who expressed interest in the results on their consent forms. The findings will be disseminated through articles in peer-reviewed journals and presentations at both national and international conferences. Additionally, results will be presented to other hospitals for sharing with their medical staff upon request. Data will be made available for reasonable requests.

## 11 Discussion

The high prevalence of PSD not only severely affects the quality of life and overall recovery of the patient, creating a vicious cycle of recovery, but also creates both mental and material stress for the patient’s caregivers (50). Currently, the treatment of PSD is based on conventional treatments, especially medication and psychotherapy, with CBT often considered a “gold standard” treatment option due to its effectiveness. However, CBT requires the involvement of a professional psychotherapist, and it is difficult for patients to achieve self-management of their illness.

Aromatherapy can address some of the shortcomings of CBT, offering potential benefits in managing PSD. It can be self-administered, promoting patient autonomy and self-care. Previous studies have shown that aromatherapy can alleviate symptoms of depression, anxiety, and sleep disturbances, but there is limited research on its specific effects on PSD. Furthermore, previous studies often rely on subjective assessment scales, lacking objective indicators related to brain function. This study aims to fill that gap by using a multimodal assessment approach, including brain function measurements through EEG and fNIRS, to enrich the objectivity of PSD assessments.

This pilot study aims to provide preliminary data on the feasibility and the efficacy of mixed herb aromatherapy inhalation for treating PSD. The feasibility outcomes, including recruitment rates, adherence rates, and participant feedback, will help identify potential challenges and inform the design of future large-scale randomized controlled trials. The insights gained from brain function assessments using EEG and fNIRS will contribute to a deeper understanding of the mechanisms underlying the therapeutic effects of aromatherapy. This study has the potential to open up new treatment avenues for PSD, offer personalized treatment options, promote patient self-management, and address practical issues such as the acceptability of aromatherapy among patients.

This study will assess the feasibility of conducting aromatherapy interventions in a clinical setting, so as to provide valuable insights for designing larger-scale randomized controlled trials. The use of culturally relevant plant powders for aromatherapy could enhance participant motivation and compliance. In this context, “culturally relevant” refers to the selection of plant powders that align with traditional Chinese medicine practices and cultural preferences, which may increase the acceptance and adherence of Chinese participants. However, this approach may limit generalizability to other populations where different forms of aromatherapy are more commonly used.

Several limitations should be noted. One limitation is the difference in the amount of intervention time between the two groups. CBT is delivered twice a week at 30 minutes per session, while aromatherapy is implemented daily at 30 minutes per session. The primary obstacle lies in the nature of the interventions: CBT sessions are typically supplemented by homework assignments that patients complete independently between sessions, whereas aromatherapy involves daily inhalation sessions to maintain consistent therapeutic effects. Harmonizing these frequencies posed significant challenges. CBT requires professional involvement and structured sessions, which cannot be easily increased in frequency without additional resources and potential patient burden. In contrast, daily aromatherapy sessions are designed for regular self-administration. This discrepancy in intervention frequencies was unavoidable within the constraints of this study. Future studies could explore ways to equalize intervention times or investigate the dose-response relationship for each treatment modality.

Another limitation is the lack of data on physical function. The course and trajectory of PSD may be affected by the trajectory of physical recovery. Since this study does not collect data on physical function, it cannot control for these confounding factors. Collecting physical function data was not feasible due to resource constraints and the study’s primary focus on mental health outcomes. Addressing both the collection of physical function data and the harmonization of intervention frequencies was not possible due to limited time, funding, and personnel. These constraints made it challenging to extend the study scope beyond mental health outcomes. Future studies should include assessments of physical function to provide a more comprehensive understanding of the factors influencing PSD. This would provide a more holistic view of PSD treatment and help control for various confounding factors.

The follow-up period is also a potential limitation. This study includes a follow-up visit after 4 weeks, considering factors such as the duration of patient hospitalization. A longer follow-up period would allow for the assessment of long-term effects of the interventions and help determine the sustainability of the treatment benefits. Future research should consider extending the follow-up period to better understand the long-term impact of aromatherapy and CBT on PSD.

Participant selection bias may be present since the study is conducted in a single rehabilitation hospital in Shanghai, limiting the generalizability of the findings to other populations or regions. The placebo effect might also influence outcomes, particularly in the aromatherapy group. Individual differences in olfactory sensitivity and preference for certain scents may affect the efficacy of aromatherapy. Lastly, psychosocial factors such as social support, stress levels, and coping mechanisms, which can influence depression outcomes, are not extensively accounted for in this study.

Additionally, the issue of cultural relevance is a notable limitation. While the use of culturally relevant plant powders may enhance compliance among Chinese participants, it may limit the generalizability of the findings to other populations where different forms of aromatherapy are preferred.

An additional consideration for future research is the cost-effectiveness of aromatherapy compared to CBT. Health economics and cost-effectiveness analyses should be conducted to determine whether aromatherapy provides a more economical treatment option without compromising efficacy. Such analyses could include direct costs (e.g., treatment expenses) and indirect costs (e.g., time off work, caregiving costs), offering a comprehensive view of the economic impact of each therapy. This is particularly relevant in healthcare settings where resource allocation and cost management are critical.

Despite these limitations, this study provides valuable preliminary data and insights that could inform future research and contribute to the development of more effective treatments for PSD. Patients with PSD are encouraged to actively participate in self-management in their daily lives, learn to apply aromatherapy to manage depressive symptoms, and form a virtuous cycle of recovery. Additionally, through EEG and fNIRS, we aim to understand more deeply how aromatherapy affects brain activity, revealing its mechanism of action in depression treatment and stimulating further research on PSD treatment.

## 12 Trial status

The research will start in April 2024 and is expected to end in April 2025. This study protocol is in its first version, registered in Chinese Clinical Trial Registry on 4 January 2024. (ChiCTR2400079491.)

## Ethics statement

This study received approval from the Ethics Committee of the Second Rehabilitation Hospital of Shanghai(approval number: 2023-20-01) and was registered with the China Clinical Trial Registration Center in January 2024 (registration number: ChiCTR2400079491). All participants are required to sign a written informed consent form in accordance with the Helsinki Declaration prior to their involvement.

## Author contributions

YL: Writing – original draft, Writing – review & editing. ZH: Writing – original draft, Writing – review & editing. KZ: Writing – original draft. XZ: Writing – original draft. YW: Writing – original draft. HX: Writing – original draft. JH: Writing – review & editing. JW: Writing – review & editing.
